# Application of complex and chemically-defined medium supplements toward cell line specific performance enhancement of biopharmaceutical production systems

**DOI:** 10.1186/1753-6561-5-S8-P25

**Published:** 2011-11-22

**Authors:** James F Babcock, Karen A Benedict, Amanda L Perlman

**Affiliations:** 1 Sheffield Center for Cell Culture Technology, Sheffield Bio-Science, A Kerry Group Business, Ithaca, NY USA

## Introduction

A series of cell-line specific complex media supplements have been developed for enhancing performance of various biopharmaceutical production systems. Created using in-house media optimization methods, these supplements are manufactured using innovative, proprietary process technology which allows for the combination of complex and/or chemically defined animal-component free media additives into a single homogeneous functional supplement. These supplements have been optimized for individual cell lines, and have proven to be suitable for use in a range of basal media. Data are presented which demonstrate the effectiveness of these optimized supplements as performance enhancers for application in CHO and SP2/0 batch culture, and as feed supplements in fed-batch systems. Preliminary data will also be presented on a parallel series of strictly chemically-defined supplements, similarly optimized for specific cell lines.

## Materials and methods

CHO data were collected using a transfected CHO-K1 line (ATCC #CCL-61), adapted to serum-free suspension culture, and engineered to constitutively express secreted embryonic alkaline phosphatase (SEAP) by means of a modified human cytomegalovirus (hCMV) promoter.

Hybridoma data were collected using a murine hybridoma suspension cell line (ATCC # CRL-1753). This line was derived from primary spleen cell cultures of animals immunized with purified human immunoglobulin. These spleen cells were then fused with Sp2/0-Ag14 myeloma cells to create the hybridoma.

Triplicate 125 ml shake-flasks contained a final medium volume of 35 ml. Duplicate spinner flasks had a working volume of 150 ml. The basal medium consisted of 100% chemically defined medium (CDM). Cultures were incubated at 37°C in 5% CO_2_. Medium supplement stock solutions were prepared at 100 g/l in the basal medium and sterilized through a 2.0 µm filter.

At appropriate points during each experiment, 1.0 ml of the culture supernatants were removed for assessing cell counts and viability. Cells were counted using a Nova BioProfile Flex automated analyzer. On the final day of each run, 500 µl of the culture supernatants were removed for SEAP or IgG analysis. Levels of SEAP in the supernatants were measured using anion-exchange HPLC on a Waters Model 2695 HPLC separations module equipped with a dual-wavelength absorbance detector. IgG was quantified using a Protein G affinity column.

## Results

**Figure 1:**
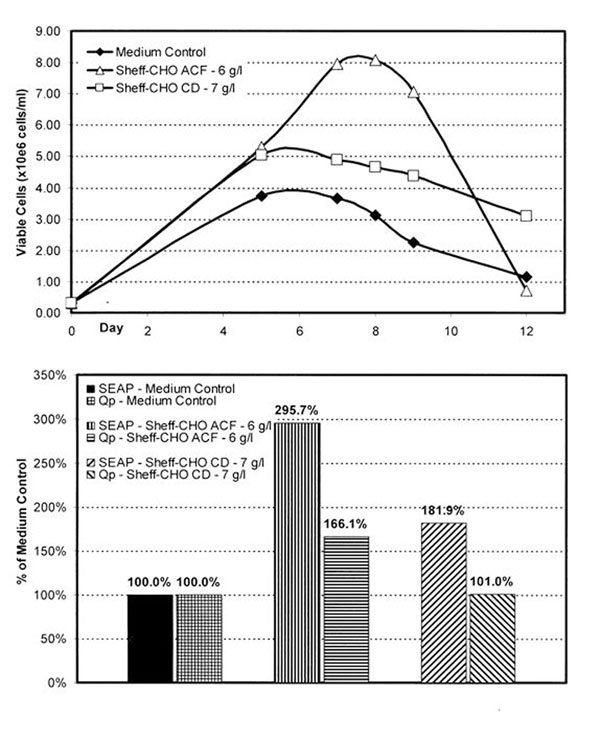
Performance comparison of defined and undefined medium supplements in CHO batch culture

Using in-house media optimization methods, both an undefined and a chemically-defined supplement for CHO cells were developed from a variety of individual components. The most favorable dosage for each supplement was determined through a series of dose-response experiments. While both supplements improved cell culture performance, the undefined supplement achieved a significantly higher peak cell density than the chemically defined supplement (Figure 1).

Both SEAP titer and specific productivity (Qp) were improved when the supplements were employed (Figure 1). As reflected in the growth data, the undefined provided a significantly greater benefit. While the titer increase for the chemically-defined supplement was substantial, there was little increase in the Qp. The significant Qp increase seen with the undefined supplement undoubtedly accounts for the higher SEAP titer obtained, and may be a function of un-characterized elements within the hydrolysate based supplement.

## Summary

Medium optimization is an integral part of biopharmaceutical process development. There is an on-going debate within the industry as to the various advantages and disadvantages of both defined and undefined media and media components. The choice of which type of system to employ is often motivated by risk mitigation with respect to consistency of performance, as weighed against the underlying goal of achieving the highest possible product titers for any given system. Defined and undefined supplementation solutions may not be mutually exclusive.

